# Supplementary material for: The Intake of Coffee Increases the Absorption of Aspirin in Mice by Modifying Gut Microbiome
Source: Pharmaceutics. 2022 Mar 30;14(4):746. doi: 10.3390/pharmaceutics14040746 (PMC9031453; doi:10.3390/pharmaceutics14040746)
Supplement: Supplementary file 1 [file pharmaceutics-14-00746-s001.zip › pharmaceutics-1641913-supplementary.pdf]

pharmaceutics

Supplementary Materials: The Intake of Coffee Increases the Absorption of Aspirin in Mice by Modifying Gut Microbiome

Jeon-Kyung Kim, Min Sun Choi, Hye Hyun Yoo and Dong-Hyun Kim

Table S1. qPCR primers for transporters.

Protein	Sequence
	Mouse
P-gp (ABCB1)	Forward; 5'-GTGGGGCAAGTCAGTTCATT-3'
	Reverse; 5'-TCTTCACCTCCAGGCTCAGT-3'
BCRP (ABCG2)	Forward; 5'-TCGCAGAAGGAGATGTGTTGAG-3'
	Reverse; 5'-CCAGAATAGCATTAAGGCCAGG-3'
Mrp4 (ABCC4)	Forward; 5'-TGCTCCTCGTCGTAAGTGTG-3'
	Reverse; 5'-TGGAGGGAGGACGATAAATG-3'
GAPDH	Forward; 5'-TGCAGTGGCAAAGTGGAGAT-3'
	Reverse; 5'-TTTGCCGTGAGTGGAGTCATA-3'
	Human
P-gp (ABCB1)	Forward; 5'-TGCTGGTCCTGAAGTTGATCTGTGAAC-3'
	Reverse; 5'-ACATTAGGCAGTGACTCGATGAAGGCA-3'
BCRP (ABCG2)	Forward; 5'-AGATGGGTTTCCAAGCGTTCAT-3'
	Reverse; 5'-CCAGTCCCAGTACGACTGTGACA-3'
Mrp4 (ABCC4)	Forward; 5'-ATATAGCCTAGATGGGCCTCTG-3'
	Reverse; 5'-GAACTTTTTCCAGCTCCTGTTC-3'
β-actin	Forward; 5'-CCATCCTGCGTCTGGACCTG-3'
	Reverse; 5'-CTCGTCATACTCCTGCTTGC-3'

Table S2. qPCR primers for gut microbiota.

Phylum	Sequence
Firmicutes	Forward; 5'-GGAGYATGTGGTTTAATTCGAAGCA-3'
	Reverse; 5'-AGCTGACGACAACCATGCAC-3'
Bacteroidetes	Forward; 5'-GTTTAATTCGATGATACGCGAG-3'
	Reverse; 5'-TTAASCCGACACCTCACGG-3'
γ,δ-Proteobacteria	Forward; 5'-GCTAACGCATTAAGTRYCCCG-3'
	Reverse; 5'-GCCATGCRGCACCTGTCT-3'
Deferribacteres	Forward; 5'-CTGACGCTGAGGTRCGAGAGC-3'
	Reverse; 5'-CCAGGTAAGGTTCTTCGGTTA-3'
TM7	Forward; 5'-GCAACTCTTTACGCCCAGT-3'
	Reverse; 5'-GCAACTCTTTACGCCCAGT-3'
Actinobacteria	Forward; 5'-TGTAGCGGTGGAATGCGC-3'
	Reverse; 5'-AATTAAGCCACATGCTCCGCT-3'
16s rRNA	Forward; 5'-TCGTCGGCAGCGTCAGATGTGTATAAGAGA-CAGGTGCCAGCMGCCGCGGTAA-3'
	Reverse; 5'-GTCTCGTGGGCTCGGAGATGTGTATAAGAGACAGGGAC-TACHVGGGTWTCTAAT-3'

Table S3. The gut microbiota composition at the phylum level in normal (NOR) mice and coffee bean extract (CBE) treated mice.

Taxon Name	Composition (%)	
	NOR	CBE
Bacteroidetes	70.78 ± 8.68	72.55 ± 11.30
Firmicutes	23.47 ± 6.66	24.63 ± 10.02
Proteobacteria	4.94 ± 2.35	2.16 ± 1.25*
Saccharibacteria_TM7	0.25 ± 0.20	0.26 ± 0.08
Cyanobacteria	0.05 ± 0.07	0.08 ± 0.05
Tenericutes	0.15 ± 0.16	0.14 ± 0.05
Deferribacteres	0.31 ± 0.29	0.12 ± 0.19

Data indicate mean S.D. *$p < 0.05$ vs. NOR group.

Table S4. The gut microbiota composition at the family level in normal (NOR) mice and coffee bean extract (CBE) treated mice.

Taxon Name	Composition (%)	
	NOR	CBE
S24-7_f	43.14 ± 8.50	48.91 ± 12.05
Prevotellaceae	17.35 ± 7.28	18.96 ± 8.34
Lachnospiraceae	16.57 ± 6.52	17.09 ± 9.56
Ruminococcaceae	5.32 ± 1.74	5.29 ± 0.93
Bacteroidaceae	5.72 ± 3.21	1.55 ± 0.65*
Rikenellaceae	2.86 ± 1.13	2.10 ± 1.28
Helicobacteraceae	3.84 ± 2.26	1.05 ± 0.76*
Lactobacillaceae	0.59 ± 0.71	1.50 ± 1.21
Desulfovibrionaceae	0.74 ± 0.68	0.67 ± 0.75
Odoribacteraceae	0.39 ± 0.24	0.27 ± 0.39

Data indicates mean S.D. *$p < 0.05$ vs. NOR group.

Table S5. The gut microbiota composition at the genus level in normal (NOR) mice and coffee bean extract (CBE) treated mice.

Taxon Name	Composition (%)	
	NOR	CBE
DQ815871_g	13.01 ± 2.56	9.21 ± 2.89*
EF602759_g	4.10 ± 1.23	5.81 ± 1.32*
Eisenbergiella	2.62 ± 0.99	7.72 ± 5.99
Alloprevotella	10.60 ± 5.14	9.38 ± 4.38
Prevotellaceae_uc	3.33 ± 6.11	4.61 ± 2.92
S24-7_f_uc	3.56 ± 1.29	4.72 ± 1.73
HM124280_g	2.15 ± 1.18	9.64 ± 4.40*
Prevotella	2.84 ± 2.27	4.20 ± 2.57
KE159538_g	4.56 ± 4.71	1.91 ± 1.27
Bacteroides	5.60 ± 3.11	1.52 ± 0.65*
HM124247_g	1.99 ± 1.05	3.45 ± 0.85*
AB606322_g	1.70 ± 0.69	2.28 ± 0.84
EF406806_g	1.36 ± 0.95	2.98 ± 0.51*
Paraprevotella	0.58 ± 1.24	0.77 ± 1.69
AY239469_g	2.54 ± 0.86	0.28 ± 0.22*

Data indicates mean S.D. *$p < 0.05$ vs. NOR group.

Table S6. The gut microbiota composition at the species level in normal (NOR) mice and coffee bean extract (CBE) treated mice.

Taxon Name	AVE ± STD	
	NOR	CBE
EF097240_s	10.34 ± 5.04	9.18 ± 4.30
Prevotellaceae_uc_s	3.33 ± 6.11	4.61 ± 2.92
S24-7_f_uc_s	3.56 ± 1.29	4.72 ± 1.73
EF603109_s	2.87 ± 0.46	2.14 ± 0.56*

EU474208_s	1.20 ± 0.90	3.40 ± 0.80*
EF406459_s	4.80 ± 0.78	1.52 ± 0.97*
AB606322_s	1.56 ± 0.64	2.15 ± 0.75
FJ880724_s	0.57 ± 1.22	0.75 ± 1.67
EF406686_s	2.26 ± 0.76	0.23 ± 0.18*
GQ157662_s	0.77 ± 0.87	6.75 ± 3.69*
EF602759_g_uc	0.70 ± 0.26	1.64 ± 0.43*
EF406536_s	3.04 ± 1.05	1.55 ± 0.65*
EU791023_s	2.60 ± 1.78	1.13 ± 0.23
EF604981_s	2.27 ± 0.82	1.24 ± 0.74*
AB599946_s	3.05 ± 1.61	0.85 ± 0.62*
EU457676_s	0.37 ± 0.25	1.12 ± 0.39*
JRMQ_s	3.58 ± 2.15	0.97 ± 0.72*
EF406712_s	1.71 ± 1.13	1.44 ± 0.30
AY239398_s	0.30 ± 0.72	1.84 ± 1.86
DQ815942_s	1.57 ± 1.56	1.94 ± 0.59

Data indicates mean S.D. *$p < 0.05$ vs. NOR group.

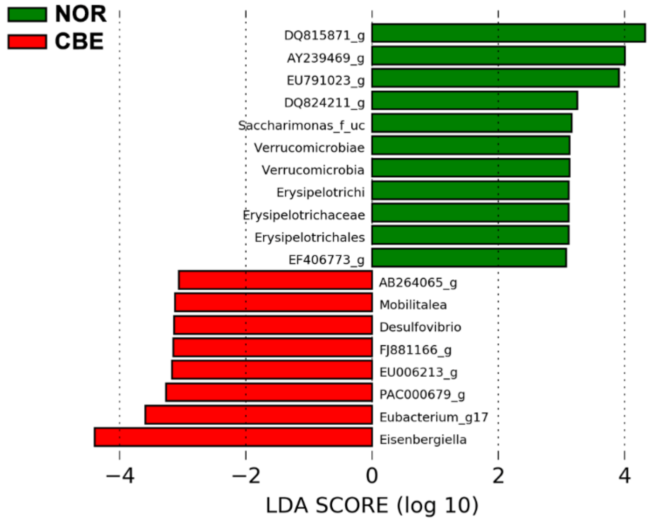

Figure S1. Effect of CBE on gut microbiota composition in mice. Gut microbiota composition was indicated in LDA score. The described strains (in species) were analyzed to the Linear Discriminant Analysis (LDA) along with effect size measurement (LEfSE) in Galaxy (http://huttenhower.sph.harvard.edu/galaxy/). It was used to discriminate significant differentially strains at each taxon level. The threshold logarithmic score set at 3 and ranked. Bacterial strains were described based on 16S rRNA sequencing data.
